# Design of a Resident Physical Fitness Data Monitoring System Based on the Sensor and Fuzzy Algorithm

**DOI:** 10.1155/2022/1742807

**Published:** 2022-08-01

**Authors:** Jun Ma, Mingxiao Ma

**Affiliations:** Xi'an Physical Education University, Xi'an, Shanxi 710068, China

## Abstract

This article first introduces the working principle and research progress of motion sensors and analyzes the advantages and disadvantages of motion tracking sensors to provide a reference for in-depth research on motion sensing technology. By combining the fuzzy judgment method to identify the kinematic parameters, this paper establishes the statistical identification of features to predict the kinematic parameters and uses the fuzzy identification method to model the human behavioral characteristics. At the same time, the article designs a set of real-time data monitoring system for athletes' real-time monitoring behavior under actual sports conditions, which is used to obtain residents' sports data. Then, based on such requirements, the overall system architecture was designed to integrate the data acquisition module, data storage and processing module, and real-time data presentation module. The motion monitor can initiate a real-time monitoring request for a specific system group, the server can monitor individual groups to add real-time individual motion status, and the system will issue an alert if the system monitors a potentially dangerous situation.

## 1. Introduction

Motion monitoring sensors are not only an important guarantee for residents' sports safety but also an important foundation for distributed wireless detection technology [1, 2]. The article first explains the working principle of the motion monitoring sensor, focuses on the latest research progress of the motion monitoring sensor, and analyzes the current advantages and disadvantages of the technology, so as to predict the development direction and prospect of the motion tracking sensor [3]. Subsequently, in view of the real-time situation of residents' physical fitness in the study area, this paper designs a set of adaptive sports data monitoring system, which can collect and monitor residents' participation, so as to provide theoretical data basis for improving the residents' fitness ability in the study area [4, 5]. First, this article studies and analyzes the needs of a real-time exercise data monitoring system. Then, according to the requirements of some system settings, the overall architecture of the system was designed, and the component structure of the system's component system and the main system module was divided into specific components, thereby determining the communication mode between the system modules and the different modules Communication protocol [6]. Subsequently, the article elaborated on the concept of sports health evaluation and the research content of the sports health evaluation model. In order to enable residents in the study area to reach the threshold of exercise time, the system provides a personalized exercise guide for each exercise behavior of the residents, and conducts the process of energy consumption [7]. Improve, put forward a health evaluation model of athletes, thereby improving the exercise effect of athletes, eliminating the adverse effects of individual physical differences in the group of athletes, thus realizing a fair group health evaluation system [8].

## 2. Materials and Methods

### 2.1. Laser Sensor Devices for Motion Monitoring

This paper designs a motion sensor model based on a laser sensor, combined with a laser sensor information mixing method, and establishes a distribution matrix model for motion monitoring behavior recognition, which can extract human motion parameters.

**Figure 1:**
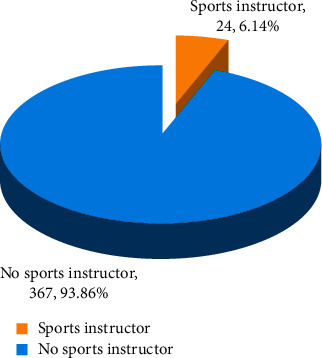
The status of physical fitness instructors of urban residents in the study area (*n* = 391).

Based on the distribution state of the laser sensor nodes of the human body motion parameter collection shown in Figure 1, a method of combining the information with multiple given parameters is selected to optimize the implementation plan. The creation of a multidimensional matrix is used to obtain some human motion parameters. The analytical model of the process matrix for obtaining human motion parameters is as follows:

(1) $$\begin{array}{c} \left[\begin{array}{c} x_{1}\left(t\right)\\ x_{2}\left(t\right)\\ \vdots \\ x_{M}\left(t\right) \end{array}\right]=\left[\begin{array}{c} \sum_{i=1}^{d}g_{1}\left(\theta_{i}\right)s_{i}\left[t-\tau_{1}\left(\theta_{i}\right)\right]\\ \sum_{i=1}^{d}g_{2}\left(\theta_{i}\right)s_{i}\left[t-\tau_{2}\left(\theta_{i}\right)\right]\\ \vdots \\ \sum_{i=1}^{d}g_{M}\left(\theta_{i}\right)s_{i}\left[t-\tau_{M}\left(\theta_{i}\right)\right] \end{array}\right]+\left[\begin{array}{c} n_{1}\left(t\right)\\ n_{2}\left(t\right)\\ \vdots \\ n_{M}\left(t\right) \end{array}\right]. \end{array}$$

The high-resolution kinematics analysis method is used to map the human kinematics data in the two-dimensional space. The estimation results of several models are described as follows:

(2) $$\begin{array}{c} x\left(t\right)=Gs\left(t\right)+n\left(t\right). \end{array}$$

Combined with the method of tracking and recognizing the related data combination of the kinematic laser sensor, the parameters of the kinematic laser sensor in the global coordination system are recognized, and the parameters of the motion space are recognized through the fuzzy prediction model. In the multibackground area, the data matrix of the human body distribution is as follows:

(3) $$\begin{array}{c} x\left(t\right)={\left[x_{1}\left(t\right)x_{2}\left(t\right)\cdots x_{M}\left(t\right)\right]}^{T}. \end{array}$$

We combine fuzzy judgment methods to identify kinematic parameters, establish statistical recognition of features to predict kinematic parameters, use fuzzy recognition methods to model human behavior characteristics, and use optimized judgment models to blur human kinematics data. The distribution of matrix upper limb movement data is as follows:

(4) $$\begin{array}{c} G=\left[\begin{array}{cccc} g_{1}\left(\theta_{1}\right) & g_{1}\left(\theta_{2}\right) & \vdots & g_{1}\left(\theta_{d}\right)\\ g_{2}\left(\theta_{1}\right) & g_{2}\left(\theta_{2}\right) & \vdots & g_{2}\left(\theta_{d}\right)\\ \ldots & \ldots & \ddots & \ldots \\ g_{M}\left(\theta_{1}\right) & g_{M}\left(\theta_{2}\right) & \vdots & g_{M}\left(\theta_{d}\right) \end{array}\right]. \end{array}$$

Based on the above analysis, the original data are used to analyze the human body parameter activity model, and a typical model is created to provide a field close to the origin of the human body parameter activity. If the parameter motion matrix of the human body satisfies *A* = *AH*, where *AH* = (*A∗*) *T*, “*∗*” represents the multiple connection between different axis numbers and “*T*” represents the transposition of each acceleration signal, combined with the laser sensor information fusion method to establish a human body detection matrix distribution model. Parametric kinematics characteristics are combined with the nature of the behavioral analysis vector sequence, so as to analyze the parameters of the kinematics model.

### 2.2. Research Methods

The exercise status of urban residents in city *Y* is the main research object, and the urban residents who often take part in physical exercises in the research area are selected as the main research subjects.

Questionnaire survey method: From December 2019 to April 2020, a questionnaire survey was conducted in 5 cities and streets (a total of 18 communities) in the study area. In the process of distributing questionnaires, we used personal interpersonal relationships to obtain the best statistical results: 457 questionnaires were mainly distributed to 5 streets, and 399 were recovered, of which 391 were valid questionnaires, with an effective rate of 97.9% (see Table 1). It should be noted that there are not many samples initially issued in this study. According to the principles of sociology, they cannot yet represent the overall situation of the population in the region, so this survey and analysis can only be used as a reference.

In this study, the reliability method of “re-testing” was adopted for the questionnaire. Subsequently, half a month after the completion of the questionnaire collection, the 47 residents who participated in the *B* Street community questionnaire survey were tested again, and the content of the questionnaire was consistent with the preliminary test, and the correlation coefficient test was *R* = 0.803 (showing a high correlation). The above data show that the content of the subject questionnaire is relatively reliable, and the results can more accurately reflect the actual situation of the survey area.

## 3. Results

### 3.1. Data Analysis of Physical Fitness of Urban Residents in the Study Area

Table 2 shows the survey statistics of environmental damage caused by physical exercise of urban residents in the study area (*n* = 391).

The road of environmental protection and sustainable development can create a socially friendly environment and save resources. The above themes all emphasize the importance of environmental protection, and in the process of physical exercise, it is also related to the protection of the ecological environment. To a certain extent, urban residents in the study area almost regard exercise as an activity close to nature. Therefore, different sports and fitness items have different effects on the environment. Based on survey data, 36.84% of fitness participants said that they may damage the ecological environment during physical exercise. Part of the reason is that some urban residents do not know enough about environmental protection, and the waste generated by them has not been effectively disposed of and managed; on the other hand, environmental damage may be the formation of self-behavior; through interviews, some participants indicated that they were participating in fitness exercises. Throwing out trash anywhere is mainly due to the unreasonable setting of trash bins. Second, throwing out trash is mainly a personal behavior habit. Another way to destroy the ecological environment is that some fitness participants will destroy them if they find their favorite animals and plants during exercise. Through interviews and investigations, it can be learned that some respondents said that they would step on the grass at will, fold flowers at will, and damage the environment while exercising. The main reasons for this phenomenon are as follows: 1. Participants have a poor understanding of nature and environmental protection and 2. their self-discipline is weak. The purpose of physical exercise for urban residents is to let most participants integrate into nature and release stress. As a sport to get close to nature, we not only need to enjoy the fun of nature while exercising and strengthening our body but also need to protect the natural environment, protect the ecological balance, and avoid any behavior that is harmful to the environment, such as littering and stepping on the lawn. The basic requirement for a qualified natural fitness enthusiast is to have good environmental protection qualities. In addition, 53.46% of the participants stated that they did not cause environmental damage. Through interviews, it was found that this type of people think that they often plan the path in advance when exercising in the wild, and they will also deal with the garbage generated, will not discard it anywhere, will not cause damage to the wild environment, and will cause bad behavior to affect the environment. The survey found that the proportion of people with higher education who damage the environment is very small. As the level of education increases, the number of people who damage the environment has also decreased, indicating that a good education is very important to environmental protection, and people with higher education have a higher quality. As a new generation of young people, we must live in harmony with nature, respect nature, and protect nature. We need to take responsibility and not destroy the natural plant environment during fitness. Do not step on the lawn. We are doing warm-up and strength exercises to strengthen our body. We should take care of the environment while enjoying your body and mind.

The areas where urban residents in the study area participate in fitness exercises are shown in Table 3:

As a necessary condition for residents to participate in fitness activities, the fitness area is the carrier, material basis, and guarantee of fitness activities. Urban residents in the study area mainly choose markets and parks as their preferred places for exercise, accounting for 31.36% of the total population. The survey found that the study area has spent a lot of time and money resources in building parks and markets in recent years. Many free sports facilities and amusement projects such as free public bicycles have also been set up in the parks and markets. In addition, the park itself has fresh air and is comfortable. Environment, etc., so it will attract a large number of residents to exercise.

Fitness exercise is a kind of exercise aimed at developing muscles, strengthening physical strength, improving physical condition, health and emotional development with bare hands or using various equipment, using specialized movements and techniques. There are many different types of sports, and everyone has their own favorite sports and exercise methods. Different exercise methods are aimed at different muscle groups, but for urban residents in the study area, no matter what exercise it is, it has its own meaning.

Fitness can make people feel happy, and it can also keep people physically and mentally healthy. There are many ways to participate in physical exercise. The urban residents in the study area participate in physical exercise in the form of participation by themselves, with family and friends, unit organizations, and community organizations. Among them, the proportion of relatives and friends participating and self-participating was the highest among all activities, 191 and 92, respectively, accounting for 48.85% and 23.53% of the total. It can be seen that the residents in the study area participate in sports mainly by relatives and friends and they are highly organized.

The situation of the instructors of sports and fitness activities of urban residents in the study area is shown in Figure 1:

Social sports instructors refer to those who participate in skill teaching, sports guidance, and organization and management of public sports activities, in addition to competitive sports and school sports. Its main task is to teach the residents to exercise and avoid injuries during sports. The abovementioned survey results show that only 6.14% of residents have participated in physical exercise under the guidance of professional sports coaches, while the remaining 93.86% of residents are only spontaneous sports, and the others are organized by untrained professionals and other organizations.

### 3.2. Design of a Real-Time Monitoring System for Residents' Sports Data

Due to the differences in user populations, the complexity of personal use characteristics, and the diversity of operating environments, real-time sports data monitoring systems must also be designed for specific purposes. The diversified and interrelated subsystems of the system are effectively organized so that each module can execute a streamlined communication and information management system to meet system requirements [9]. This chapter examines the requirements of the sports data real-time monitoring system, proposes a blueprint for the system architecture, and describes and defines the communication mechanism between the various components of the system in detail, laying a foundation for the further expansion of the system.

Consistent with the above requirements analysis, the system architecture can be divided into four parts. The overall architecture of the sports data real-time monitoring system includes the following:

#### 3.2.1. Data Extraction Module

This component is mainly composed of data extraction tools and communication modules. The data extraction device is worn on the waist of the athlete and is responsible for collecting the sports behavior data generated by the 3D acceleration sensor during the movement, and then sending it to the database station through the data communication module [10].

#### 3.2.2. Data Receiving and Transmission Component

This component is mainly composed of a database station and a communication module. The database station is placed in the activity monitoring area and is mainly used to receive data from the data collection equipment worn by the athletes, and then transmit the data to the system and component data analysis for the next step [11].

#### 3.2.3. Data Analysis and Processing Module

This component is mainly composed of system communication processing module, data storage module, and planned task management module, which is the core component of the system [12]. It connects and controls other system submodules to jointly complete system-wide functions. This feature will be discussed later.

#### 3.2.4. Data Display Function

**Figure 2:**
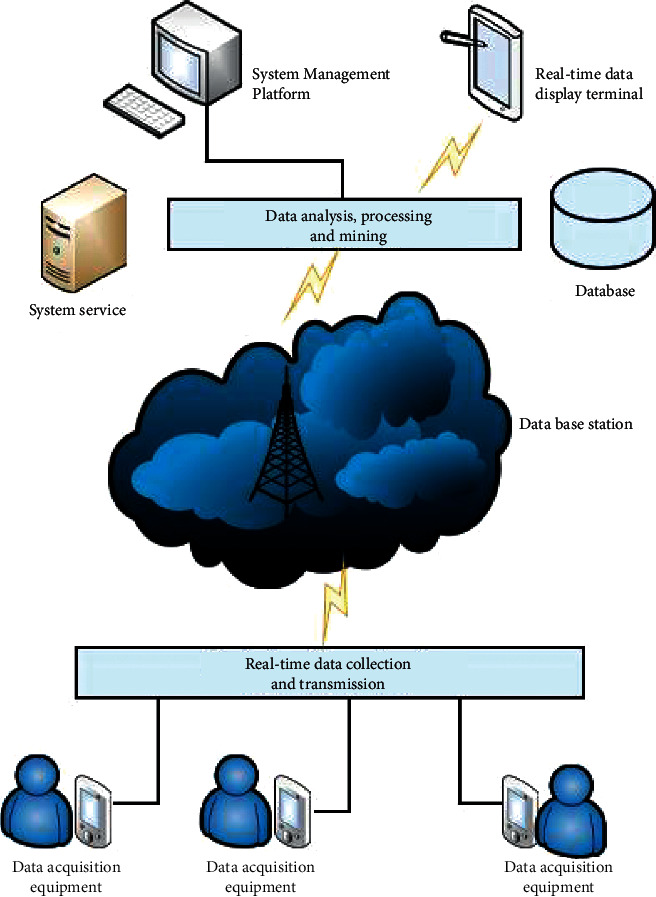
Overall architecture of the real-time monitoring system for exercise data.

This function is mainly composed of data communication components and data display software. The data display software is mainly provided on two platforms, the mobile terminal and the Web management terminal [13]. Among them, the real-time monitoring of sports data is mainly on the mobile terminal platform. Through the software that displays real-time data on the mobile terminal, the motion monitor can initiate a real-time monitoring request for a specific system group [14]. The server can monitor individual groups to add real-time personal exercise status. If the system monitors a possible dangerous situation, the system will issue an alarm to remind the monitor to take appropriate treatment as soon as possible to achieve the purpose of risk avoidance. The overall architecture of the real-time exercise data monitoring system is shown in Figure 2.

**Figure 3:**
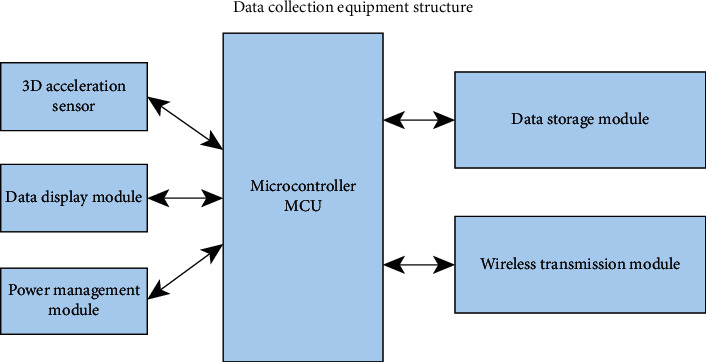
The structure of the data acquisition equipment.

Athletes wear data acquisition devices when participating in sports, which can monitor the number of steps and energy consumption of the human body in real time. According to the analysis and design of the system requirements, the data acquisition equipment consists of a microcontroller (MCU), a 3D acceleration sensor real-time data acquisition system, a data display module, an energy management module, a data storage module and a wireless transmission module, as shown in Figure 3.

#### 3.2.5. Microcontroller (MCU)

MCU microcontroller is the basic module of data retrieval equipment, which has the function of connecting and controlling other modules of the equipment. It is mainly responsible for controlling the sensor to collect the raw data of human movement, identifying and analyzing, and using the step counting algorithm to obtain the number of movement steps. The MCU creates a wireless communication protocol that conforms to the communication standard between the data retrieval device and the database station. In addition, it has created an energy management strategy for data collection equipment to achieve short-term energy consumption and energy saving.

#### 3.2.6. 3D Acceleration Sensor

The data acquisition device is worn on the waist of the athlete to monitor sports data. The 3D acceleration sensor can record the vertical acceleration of the waist. The change of vertical acceleration can effectively identify the number of steps in the movement, and send the acceleration data executed during the movement to the MCU to calculate the number of steps of the athlete.

#### 3.2.7. Data Display Mode

The data display module is mainly used to display the number of exercise steps tracked by the device to obtain data, and it can also display the remaining power of the device.

#### 3.2.8. Power Management

Data acquisition equipment must be equipped with lithium batteries for power supply. The power management module is more used to manage the power transmission mode of the battery charging and the power supply strategy of the device to capture data of different working modes such as (real-time monitoring and static sleep).

#### 3.2.9. Data Storage Module

Real-time monitoring of data acquisition equipment will generate a large amount of exercise data, which needs to be temporarily stored in the data storage module before being sent to the data base station. This requires the data storage module to realize the storage and replacement strategy of exercise data.

#### 3.2.10. Wireless Transmission Module

The wireless transmission module sends the movement data of the monitoring station to the database for transmission through the 2.4 GHz wireless transmission protocol.

## 4. Conclusion

This paper first establishes a comprehensive objective function, the system uses constraint function and random chance control program model, and uses LINGO software to solve the calculation. Second, the calculation results are analyzed from the design schemes and optimization schemes executed under three different confidence levels. In each scenario, the confidence level is selected by the economic priority scenario. The area with a *qz* value of 0.99 is the design criterion for the future energy structure restoration program. Finally, the article conducted a research on the health status of residents in the study area and designed a health data monitoring system. In view of the decline in residents' physical fitness year by year, the State Council issued guidelines on strengthening residents' physical exercise in order to improve residents' physical fitness. In order to respond to the call for guidance and address the needs of community residents' fitness monitoring and health assessment, this article is based on the Ministry of Education's key laboratory, designed and implemented a residents' health assessment platform and an online fitness service platform, thus making a unique contribution to strengthening the fitness of residents in the study area.

## Figures and Tables

**Table 1:** The issuance and recovery of the questionnaire.

Street name	Number of issued (copies)	Recycling number (parts)	Recovery rate (%)	Effective number (parts)	Efficient (%)
Street *A*	94	83	88.3	81	97.7
*B* street	88	78	88.5	77	98.6
*C* street	90	78	86.6	77	98.6
*D* street	92	81	88.1	80	98.7
*E* street	91	78	85.6	76	97.5
(Total)	457	399	87.4	391	97.9

**Table 2:** Survey statistics of environmental damage caused by physical fitness exercises of urban residents in the study area (*n* = 391).

Whether it damages the environment	Yes	No	Uncertain
	Proportion of people (%)	Proportion of people (%)	Proportion of people (%)
Total people	144/36.84	209/53.46	38/9.70
Junior high school	17/4.34	0/0.00	0/0.00
High school	73/18.66	4/1.03	0/0.00
College	39/9.98	48/12.26	9/2.31
Undergraduate	12/3.09	152/38.86	27/6.90
Postgraduate and above	3/0.75	5/1.29	2/0.52

**Table 3:** Table of places where urban residents participate in fitness exercises in the study area (*n* = 391).

Activity venue	Number of people	Percentage	Sort
Public stadium	71	18.17	2
Squares and parks	124	31.36	1
School stadium	0	0	7
Street, alley	57	14.56	3
Unit sports facilities	38	9.72	5
Residential green space	48	12.26	4
Charging place	35	8.94	6
Other	18	4.62	8

## Data Availability

The data used to support the ﬁndings of this study are available from the corresponding author upon request.

## References

[1] Milea L. (2015). Detection and tele-replication of human hand motions by a robotic hand. *American Journal of Aerospace Engineering*.

[2] Nakamoto H., Ootaka H., Tada M., Hirata I., Kobayashi F., Kojima F. (2016). Stretchable strain sensor with anisotropy and application for joint angle measurement. *IEEE Sensors Journal*.

[3] Ma Z., Cai S., Mao N., Yang Q., Feng J., Wang P. (2018). Construction quality management based on a collaborative system using bim and indoor positioning. *Automation in Construction*.

[4] Verstegen L., Houkes W., Reymen I. (2019). Configuring collective digital-technology usage in dynamic and complex design practices. *Research Policy*.

[5] Marras W. S., Fathallah F. A., Miller R. J., Davis S. W., Mirka G. A. (1992). Accuracy of a three-dimensional lumbar motion monitor for recording dynamic trunk motion characteristics. *International Journal of Industrial Ergonomics*.

[6] Geng Y., Chen J., Fu R., Bao G., Pahlavan K. (2016). Enlighten wearable physiological monitoring systems: on-body RF characteristics based human motion classification using a support vector machine. *IEEE Transactions on Mobile Computing*.

[7] Sun M., Jiang Y., Liu Q., Liu X. (2019). An auto-calibration approach to robust and secure usage of accelerometers for human motion analysis in FES therapies. *Computers, Materials & Continua*.

[8] Yoo D. H. (2020). Effects of dance sports and yoga program on body composition, physical fitness, blood lipids and liver function Indicator in the elderly. *Exercise Science*.

[9] Tai K. T., Tai J. C. (2006). Dynamic calibration of pan-tilt-zoom cameras for traffic monitoring. *IEEE Transactions on Systems, Man and Cybernetics, Part B (Cybernetics)*.

[10] Zhou X., Zhu M., Pavlakos G., Leonardos S., Derpanis K. G., Daniilidis K. (2019). MonoCap: monocular human motion capture using a CNN coupled with a geometric prior. *IEEE Transactions on Pattern Analysis and Machine Intelligence*.

[11] Wang P., Liu H., Wang L., Gao R. X. (2018). Deep learning-based human motion recognition for predictive context-aware human-robot collaboration. *CIRP Annals*.

[12] Ruscello B., Esposito M., Lunetta G., D’ottavio P. R., D’ottavio S. (2020). Gender differences in instep soccer kicking biomechanics, investigated through a 3D human motion tracker system. *The Journal of Sports Medicine and Physical Fitness*.

[13] Chen L., Sun H., Zhao W., Yu T. (2021). Robotic arm control system based on AI wearable acceleration sensor. *Mathematical Problems in Engineering*.

[14] Zhao L., Zhao Y., Wang X. (2021). Athleteʼs physical fitness prediction model algorithm and index optimization analysis under the environment of AI. *Mathematical Problems in Engineering*.

